# c-Abl-mediated Drp1 phosphorylation promotes oxidative stress-induced mitochondrial fragmentation and neuronal cell death

**DOI:** 10.1038/cddis.2017.524

**Published:** 2017-10-12

**Authors:** Lujun Zhou, Qiang Zhang, Peng Zhang, Lei Sun, Can Peng, Zengqiang Yuan, Jinbo Cheng

**Affiliations:** 1 State Key Laboratory of Brain and Cognitive Sciences, Institute of Biophysics, Chinese Academy of Sciences, Beijing 100101, China; 2 Sino-Danish College, University of Chinese Academy of Sciences, Beijing 100049, China; 3 Center for Biological Imaging, Institute of Biophysics, Chinese Academy of Sciences, Beijing 100101, China; 4 The Brain Science Center, Beijing Institute of Basic Medical Sciences, Beijing 100850, China; 5 Center of Alzheimer’s Disease, Beijing Institute for Brain Disorders, Beijing, China

## Abstract

Oxidative stress-induced mitochondrial dysfunction and neuronal cell death have important roles in the development of neurodegenerative diseases. Dynamin related protein 1 (Drp1) is a critical factor in regulating mitochondrial dynamics. A variety of posttranslational modifications of Drp1 have been reported, including phosphorylation, ubiquitination, sumoylation and *S*-nitrosylation. In this study, we found that c-Abl phosphorylated Drp1 at tyrosine 266, 368 and 449 *in vitro* and *in vivo*, which augmented the GTPase activity of Drp1 and promoted Drp1-mediated mitochondrial fragmentation. Consistently, c-Abl-mediated phosphorylation is important for GTPase activity of Drp1 and mitochondrial fragmentation. Furthermore, we found that Drp1 phosphorylation mediated by c-Abl is required for oxidative stress-induced cell death in primary cortical neurons. Taken together, our findings reveal that c-Abl-Drp1 signaling pathway regulates oxidative stress-induced mitochondrial fragmentation and cell death, which might be a potential target for the treatment of neurodegenerative diseases.

Oxidative stress-induced mitochondrial dysfunction and neuronal cell death have been implicated as crucial steps in neurodegenerative diseases.^1, 2, 3, 4, 5^ Mitochondria are highly dynamic organelles with continuous fission and fusion that are regulated by several GTPases, such as dynamin-related protein (Drp1) for fission and mitofusin 2 (MFN2) for fusion.^6^ Impaired balance of fission and fusion events causes mitochondrial dysfunction and leads to human neurological diseases.^7, 8, 9, 10^ Recent studies have proved that inhibition of excessive mitochondrial fission executes a protective role against neurotoxicity.^11, 12^

Upon oxidative stress, Drp1 is recruited to fission sites on the mitochondrial outer membrane and initiates mitochondrial fragmentation.^13^ It has been reported that Drp1 activity is tightly regulated by posttranslational modifications, including phosphorylation, ubiquitination, sumoylation, *S*-nitrosylation and so on.^14, 15, 16, 17, 18, 19, 20^ For example, phosphorylation of Drp1 at Ser^637^ by cAMP-dependent kinase (PKA) could decrease the GTPase activity of Drp1, resulting in reduced mitochondrial fission.^14^ Ubiquitination of Drp1 mediated by E3 ligase Parkin leads to the degradation of Drp1 and thus inhibits mitochondrial fission.^16^ Sumoylation of Drp1 protects Drp1 from degradation and induce excess mitochondrial fragmentation.^19^ Accumulated studies have also demonstrated that aberrant modification of Drp1 leads to dysregulation of mitochondria associated with neuronal injury and neurodegenerative diseases.^3, 16, 18, 21, 22^

Previously we have demonstrated that c-Abl has an important role in oxidative stress-induced neuronal cell death.^23, 24^ Ko *et al.*^25^ and Imam *et al.*^26^ have reported that c-Abl-mediated Parkin phosphorylation inhibited its E3 ligase activity that led to the neurotoxic accumulation of Parkin’s substrates. It has also been reported c-Abl phosphorylated *α*-synuclein in Parkinson’s disease (PD).^27^ The above findings link c-Abl tyrosine kinase to oxidative stress-induced neuronal cell death and the development of neurodegenerative diseases. Interestingly, c-Abl could translocate to the mitochondria in response to oxidative stress and regulated oxidative-induced neuronal cell death,^28^ suggesting that c-Abl might be involved in mitochondrial dynamics in response to oxidative stress. However, the molecular mechanism underlying c-Abl-regulated mitochondrial homeostasis remains largely elusive under stress conditions.

In this study, we demonstrated that c-Abl was involved in the regulation of mitochondrial morphology *in vivo*. Interestingly, we found that Drp1 was a substrate of c-Abl kinase and ectopic expression of c-Abl increased Drp1’s mitochondrial localization and GTPase activity. Furthermore, c-Abl-mediated phosphorylation increases GTPase activity of Drp1 without affecting its stability and mitochondrial localization. Lastly, c-Abl expression induced neuronal cell death, which could be abrogated by Drp1 knockdown. In addition, Y (tyrosine, identified tyrosine phosphorylation site by c-Abl) to F (phenylalanine) mutants of Drp1 reduced oxidative stress-induced cell death. Taken together, our findings identified that c-Abl-Drp1 signaling regulates mitochondrial morphology and neuronal cell death in response to oxidative stress, with implication of a new avenue for the treatment of oxidative stress-induced brain diseases by targeting c-Abl–Drp1 pathway.

## Results

### c-Abl is involved in the regulation of mitochondrial morphology *in vivo*

**Figure 1:**
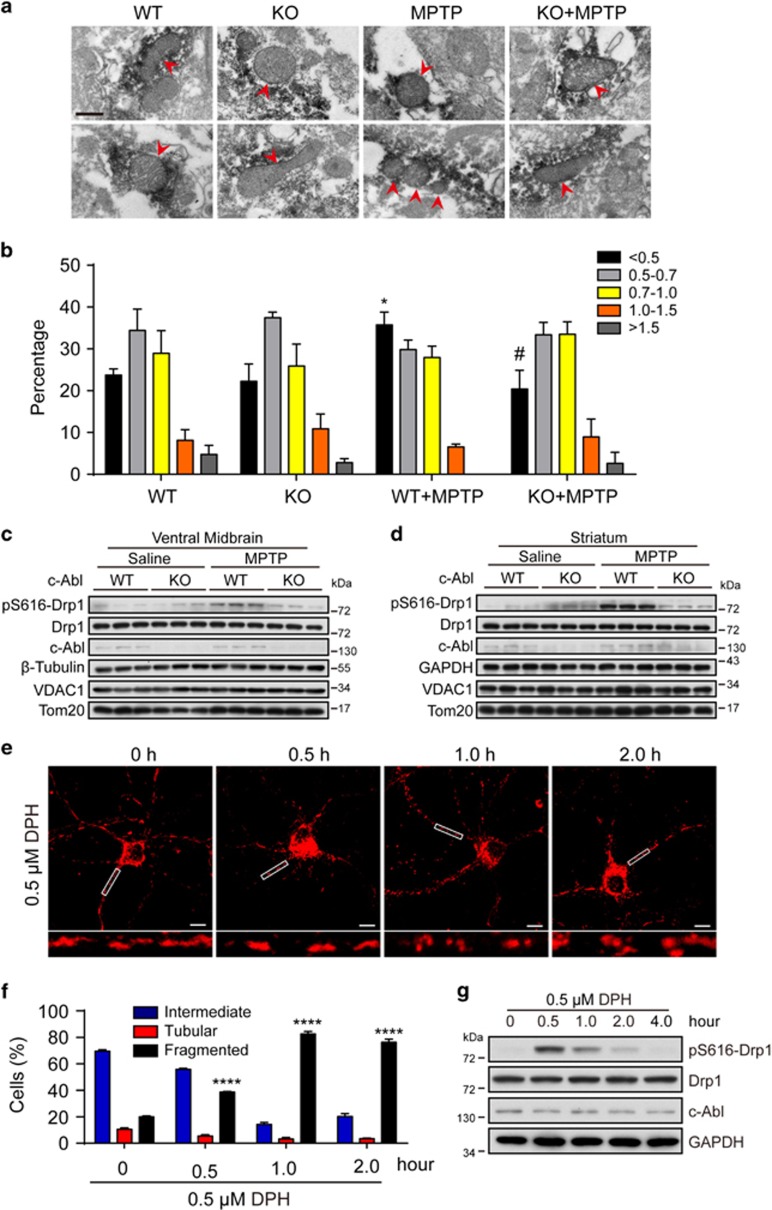
c-Abl is involved in the regulation of mitochondrial morphology *in vivo.* (**a**) WT and c-Abl KO littermate mice were treated with saline or MPTP (four i.p. injections of 20 mg/kg at 2 h intervals). At 2 h after total injections, immuno-EM was performed as described in Materials and Methods section. Two representative images (upper and lower) were picked for each group. Arrows indicate tyrosine hydroxylase-positive area containing mitochondria in SN. Scale bars, 0.5 *μ*m. (**b**) Mitochondrial length was measured and divided into different categories of <0.5, 0.5–0.7, 0.7–1.0, 1.0–1.5 and >1.5 *μ*m. Quantification of mitochondrial length is shown as percentages of total mitochondria (at least 50 mitochondria were measured per mouse). (two-way ANOVA, *P<0.05 compared with the WT group, #*P*<0.05 compared with the WT+MPTP group, *n*=3 mice.). (**c**) Striatal tissue or (**d**) ventral midbrain was collected from WT and c-Abl KO littermate mice 2 h after treatment with saline or MPTP and subjected to immunoblotting using the indicated antibodies. (**e**) Primary cortical neurons were immunostained with the anti-Tom20 antibody after 0.5 *μ*M DPH treatment for 0, 0.5, 1 and 2 h, respectively, followed by confocal microscopy. Scale bars, 10 *μ*m. (**f**) Quantification of mitochondrial morphology from panel (**e**). (two-way ANOVA, fragmented: ****P<0.0001, compared with 0 h group; *n*=3 slides). (**g**) Lysates of primary cortical neurons treated with 0.5 *μ*M DPH for the indicated times were immunoblotted with the indicated antibodies

c-Abl has been implicated in the pathogenesis of neurodegeneration, in which mitochondria are usually dysregulated.^29^ The neurotoxin MPTP (1-methyl-4-phenyl-1, 2, 3, 6-tetrahydropyridine) has been widely used to establish the murine model of PD.^30^ To explore the role of c-Abl in mitochondrial dynamics, c-Abl^flox/flox^ mice were crossed with CamKII*α*-iCre transgenic mice to generate the mice with c-Abl deletion in neurons. Wild-type (WT, c-Abl^flox/flox^) and c-Abl cKO (c-Abl^flox/flox^; CamKII *α*-iCre^+/-^) mice were then treated with saline or MPTP (four intraperitoneal injections of 20 mg/kg at 2 h intervals). 2 h after MPTP treatment, the length of mitochondria was assessed by immuno-electron microscopy at tyrosine hydroxylase (TH)-positive neurons from substantia nigra (SN) and striatum (Figures 1a and b and Supplementary Figure S1A). Quantitative morphological analysis demonstrated that mitochondrial length significantly shifted from elongated to a fragmented phenotype induced by MPTP treatment. A total of 35.72±5.32% of mitochondria exhibited a length of <0.5 *μ*m in the WT+MPTP group compared with 23.37±2.55% in the WT+Saline group. The mitochondrial length <0.5 *μ*m is about 22.24±7.18% in c-Abl cKO SN, indicating that c-Abl cKO has no effect on the basal mitochondrial length. Interestingly, there is about 20.40±7.75% of mitochondria in the c-Abl cKO+MPTP group that displayed fragmented phenotype (length<0.5 *μ*m), indicating that c-Abl cKO mitigated excessive mitochondrial fragmentation induced by MPTP and arguing that c-Abl might have an important role in the regulation of mitochondrial morphology. Overall, mitochondrial length was decreased by MPTP treatment in the WT+MPTP group but not in the c-Abl cKO+MPTP group (Supplementary Figures S1B and C). In addition, we found that Drp1^Ser616^ phosphorylation levels, a molecular marker for mitochondrial fission,^1^ was strikingly induced by MPTP treatment and markedly decreased in c-Abl KO SN and striatum (Figures 1c and d). In another way, we examined whether pharmacological activation of c-Abl could induce mitochondrial fission. DPH (5-(1, 3-diaryl-1H-pyrazol-4-yl) hydantoin), a small cell-permeable molecule, has been reported as a c-Abl activator.^31^ Treatment of primary cortical neurons with DPH resulted in a dramatically increased percentage of cells with fragmented mitochondria at 1–2 h (Figures 1e and f). Interestingly, in time-course experiment, Drp1^Ser616^ phosphorylation was induced within 0.5 h after DPH treatment in primary neurons (Figure 1g) and SH-SY5Y cells (a human neuroblastoma cell line) (Supplementary Figure S1D). Taken together, these data suggest that c-Abl is responsible for the excessive mitochondrial fragmentation in MPTP-induced PD model.

### c-Abl interacts with and phosphorylates Drp1

**Figure 2:**
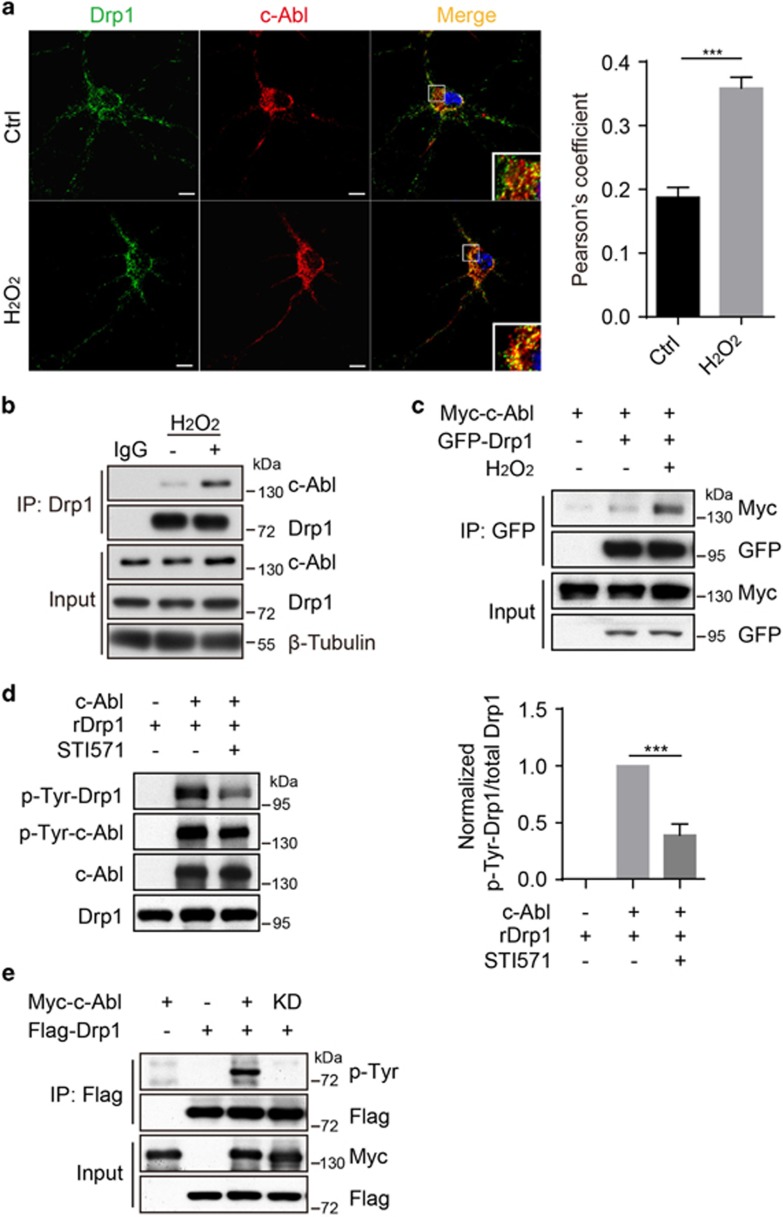
c-Abl interacts with and phosphorylates Drp1. (**a**) Left panel: primary cortical neurons were immunostained with the anti-Drp1 and anti-c-Abl antibodies after 100 *μ*M H_2_O_2_ treatment or not for 0.5 h, followed by confocal microscopy. Right panel: Pearson’s correlation coefficient (Student’s *t*-test, ***P<0.001, *n*=4). (**b**) Lysates of primary cortical neurons with or without 100 *μ*M H_2_O_2_ treatment for 0.5 h were immunoprecipitated with the anti-Drp1 antibody followed by immunoblotting with the anti-c-Abl antibody. (**c**) Lysates from HEK-293T cells transfected with expression plasmids Myc-c-Abl and GFP-Drp1 with or without 1 mM H_2_O_2_ treatment for 0.5 h were immunoprecipitated with anti-GFP antibody and subjected to immunoblot with the anti-Myc antibody. (**d**) Left panel: c-Abl kinase was subjected to an *in vitro* kinase assay using the recombinant full-length His-sumo-Drp1 (rDrp1) as a substrate with or without 10 *μ*M STI571 treatment. Phosphorylation was analyzed by immunoblotting with anti-phospho-tyrosine antibody. Right panel: The normalized levels of p-Tyr-Drp1. (Student’s *t*-test, ***P<0.001, *n*=3). (**e**) Lysates from HEK 293T cells transfected with the Flag-Drp1 and Myc-c-Abl-WT or Myc-c-Abl-KD (kinase dead) expression plasmids were immunoprecipitated with anti-Flag antibody, then immunoblotted with anti-phospho-tyrosine antibody

As c-Abl is a tyrosine kinase and the mitochondrial fragmentation is significantly reduced by c-Abl KO, we next tested the possibility that Drp1 was a direct substrate of c-Abl in the regulation of mitochondrial dynamics. Here we found that hydrogen peroxide (H_2_O_2_) treatment could lead to significant colocalization between Drp1 and c-Abl in primary neurons (Figure 2a). Then co-immunoprecipitation was used to define the interaction between Drp1 and c-Abl and whether the interaction is influenced by H_2_O_2_ treatment. As expected, Drp1 showed a stronger interaction with c-Abl when cells were treated with H_2_O_2_ in primary neurons (Figure 2b) or HEK 293T cells (Figure 2c). Furthermore, we found that recombinant His-sumo-fused Drp1 protein was directly phosphorylated by c-Abl by using an *in vitro* kinase assay, and this phosphorylation could be attenuated by STI571, a selective inhibitor of c-Abl (Figure 2d). Meanwhile, *ex vivo* experiment demonstrated that WT, but not kinase dead form of c-Abl, phosphorylates Drp1 in HEK 293T cells by using the pan-phospho-Tyrosine antibody (Figure 2e). Interestingly, we found that Drp1 phosphorylation happens in both cytoplasm and isolated mitochondria and c-Abl overexpression increases tyrosine phosphorylation of mitochondrial Drp1 (Supplementary Figure S2A). Together, these data suggest that Drp1 is a direct substrate of c-Abl.

**Figure 3:**
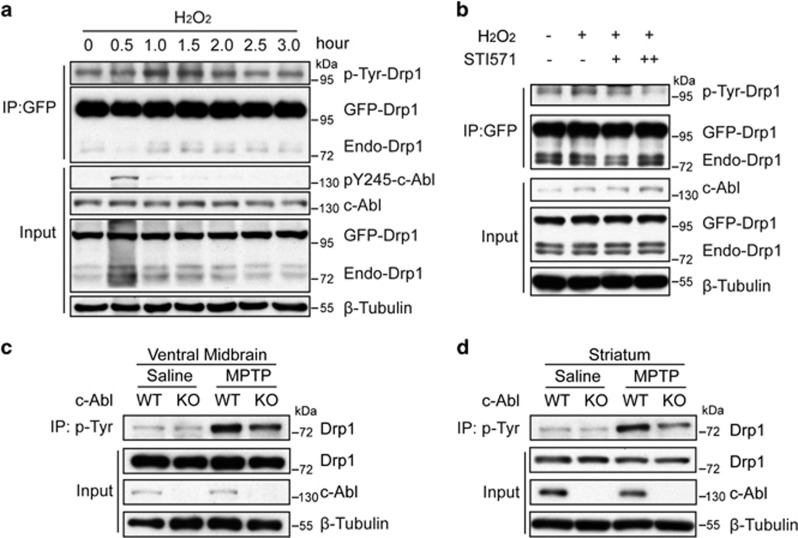
c-Abl activation promotes tyrosine phosphorylation of endogenous Drp1. (**a**) Lysates of SH-SY5Y cells with 1 mM H_2_O_2_ treatment for the indicated times were immunoprecipitated with GFP antibody and subjected to immunoblot with the indicated antibodies. (**b**) Lysates of SH-SY5Y cells with 1 mM H_2_O_2_ treatment for 1 h, along with or without STI571 treatment, were immunoprecipitated with GFP antibody and subjected to immunoblot with the indicated antibodies. (**c** and **d**) Mice were treated in the same way as in Figure 1a. Ventral midbrain (**c**) and striatal tissues (**d**) were collected and subjected to immunoprecipitation using an anti-phospho-tyrosine antibody followed by immunoblotting with the indicated antibodies

To investigate the Drp1 tyrosine phosphorylation in cells, GFP-tagged Drp1 cDNA was stably introduced into SH-SY5Y cells via the retro-virus infection. Drp1 tyrosine phosphorylation was induced by H_2_O_2_ treatment for 1 h, peaked at 1–1.5 h and declined after 2 h. We also observed that the activation of c-Abl was induced within 0.5 h and declined afterwards (Figure 3a). Pretreatment with STI571 reduced the levels of H_2_O_2_-induced Drp1 tyrosine phosphorylation (Figure 3b). Importantly, c-Abl KO in neurons significantly reduced MPTP-induced tyrosine phosphorylation of Drp1 in PD model (Figures 3c and d). Together, our data implicate that the tyrosine phosphorylation level of Drp1 is regulated by c-Abl upon oxidative stress.

### c-Abl phosphorylates Drp1 at Y266, Y368 and Y449

**Figure 4:**
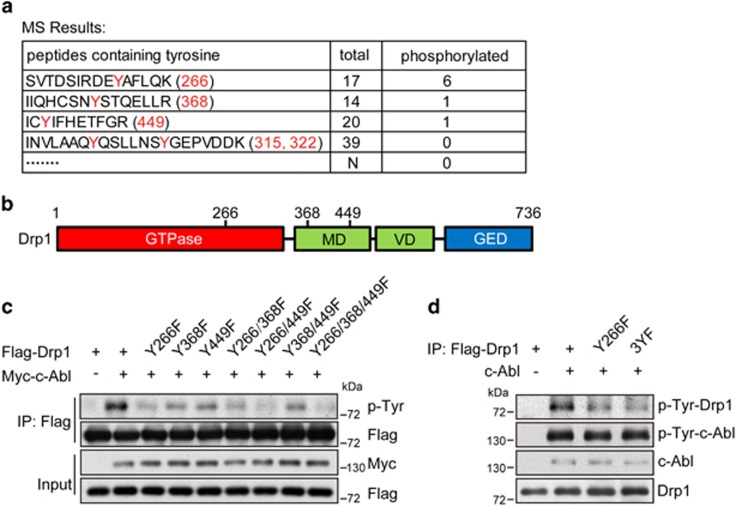
c-Abl phosphorylates Drp1 at Y266, Y368 and Y449. (**a**) Phosphorylation reactions from Figure 2d were subjected to SDS-PAGE followed by Coomassie Blue staining. The band corresponding to Drp1 was excised from the gel and digested with trypsin. Phosphorylation sites were mapped by microcapillary liquid chromatography-MS/MS, with 92.8% coverage of the Drp1 amino-acid sequence. Three phosphorylated tyrosine sites were identified. (**b**) Schematic of three phosphorylation sites in the Drp1 protein. (**c**) Lysates from HEK 293T cells transfected with Flag-Drp1 WT or other Drp1 mutants as indicated and Myc-c-Abl WT expression plasmids were immunoprecipitated with anti-Flag antibody and analyzed by immunoblotting anti-phospho-tyrosine antibody. (**d**) Immunoprecipitates of Flag-Drp1 WT and Y226F and 3YF mutants transfected in HEK 293T cells were subjected to *in vitro* kinase assay with c-Abl kinase. Phosphorylation reactions were analyzed by immunoblotting with anti-phospho-tyrosine antibody

To further define the phosphorylation sites of Drp1 by c-Abl, *in vitro* kinase assay and mass spectrometric analysis was performed, and three tyrosine sites were identified (Figure 4a). Among these sites, Y266 is located within the GTPase domain and Y368 and Y449 are localized in the middle domain of Drp1 protein (Figure 4b). Then site-directed mutagenesis technique was used to convert the tyrosine to phenylalanine, including single-site mutations, two-site combination mutations and three-site mutations. In cells, we found that there is a dramatic decrease of c-Abl-induced tyrosine phosphorylation of mutant Drp1, including the individual and combination mutation of Drp1 Y266F, Y368F and Y449F (Figure 4c). We observed that mutant Drp1 Y266F showed the lowest tyrosine compared with other mutations, indicating that Y266 might be the major phosphorylation site mediated by c-Abl (Figures 4c and d). Taken together, these results indicate that Y266, Y368 and Y449 of Drp1 are the sites phosphorylated by c-Abl.

### c-Abl-induced Drp1 phosphorylation upregulates its GTPase activity

**Figure 5:**
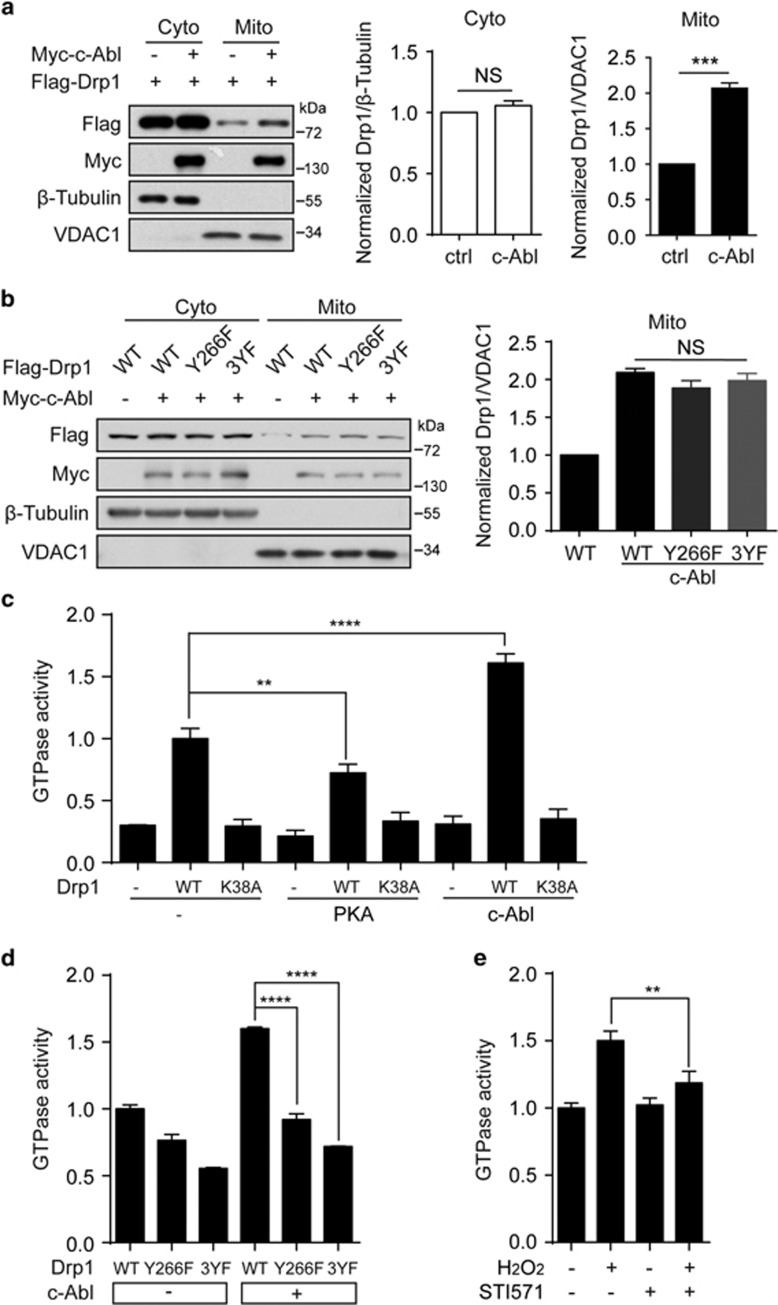
c-Abl-mediated Drp1 phosphorylation increases the GTPase activity of Drp1. (**a**) Left panel: Lysates of cytosolic and mitochondrial fractions of HEK 293T cells transfected with Flag-Drp1, with or without Myc-c-Abl, were immunoblotted with the indicated antibodies. Middle panel: The normalized levels of Drp1 in cytosolic fraction. (Student’s *t*-test, *P*=0.2, *n*=3). Right panel: The normalized levels of Drp1 in mitochondrial fraction. (Student’s *t*-test, ***P<0.001, *n*=3). (**b**) Left panel: Lysates of cytosolic and mitochondrial fraction of HEK 293T cells transfected with Flag-Drp1 WT and other mutants, with or without Myc-c-Abl, were immunoblotted with the indicated antibodies. Right panel: The normalized levels of Drp1 in mitochondrial fraction. (One-way ANOVA, *P*>0.05, *n*=3). (**c**) Immunoprecipitated Drp1 WT or K38A transfected with vector or c-Abl or PKA in HEK 293T were subjected to GTPase assay. Drp1 K38A and PKA group were used as negative and positive controls, respectively (one-way ANOVA, **P<0.01, ****P<0.0001, *n*=3). (**d**) Immunoprecipitated Drp1 WT or mutants transfected with or without c-Abl in HEK 293T were subjected to GTPase assay. (one-way ANOVA, ****P<0.0001, *n*=3). (**e**) SH-SY5Y cells were untreated or treated with 200 *μ*M H_2_O_2_ for 4 h, with or without 5 *μ*M STI571. Then immunoprecipitated Drp1 were subjected to GTPase assay. (one-way ANOVA, **P<0.01, *n*=3). NS, not significant

To initiate mitochondrial fission, Drp1 is first recruited from cytoplasm to the outer mitochondrial membrane (OMM). Hence, Drp1 molecules become dimers through a self-assembling process and forms a ring-like multimeric structure on the prospective OMM fission sites, followed by GTP hydrolysis-driven conformational changes and lead to membrane severing and final mitochondrial division.^32^ To investigate how c-Abl is involved in Drp1-mediated mitochondrial fission, we first examined the effect of c-Abl on the translocation of Drp1. The results showed that overexpression of c-Abl indeed promoted the translocation of Drp1 WT onto mitochondrial membrane (Figure 5a). However, Drp1 Y266F or 3YF did not block this effect (Figure 5b), indicating that the identified Drp1 phosphorylation sites are not directly related to its mitochondrial translocation. Next, we detected the effect of c-Abl on the self-assembly of Drp1, including intramolecular and intermolecular interactions. As shown in Supplementary Figures S3A and B, c-Abl failed to alter these interval interactions through co-immunoprecipitation assay. We next investigated whether c-Abl-mediated phosphorylation affected the GTPase activity of Drp1. To this end, we ectopically expressed Flag-Drp1 WT and mutant Drp1 with or without Myc-c-Abl. As shown in Figures 5c and d, overexpression of c-Abl induced a 1.6-fold increase in the GTPase activity of WT Drp1 but not mutant ones. Importantly, the increased GTPase activity of Drp1 induced by H_2_O_2_ treatment was attenuated by STI571 in SH-SY5Y cells (Figure 5e). Taken together, these data suggested that c-Abl promotes Drp1 mitochondrial translocation independent of identified phosphorylation sites and c-Abl-induced phosphorylation enhances Drp1 GTPase activity.

### c-Abl mediated Drp1 phosphorylation is associated with mitochondrial fragmentation and neuronal cell death

**Figure 6:**
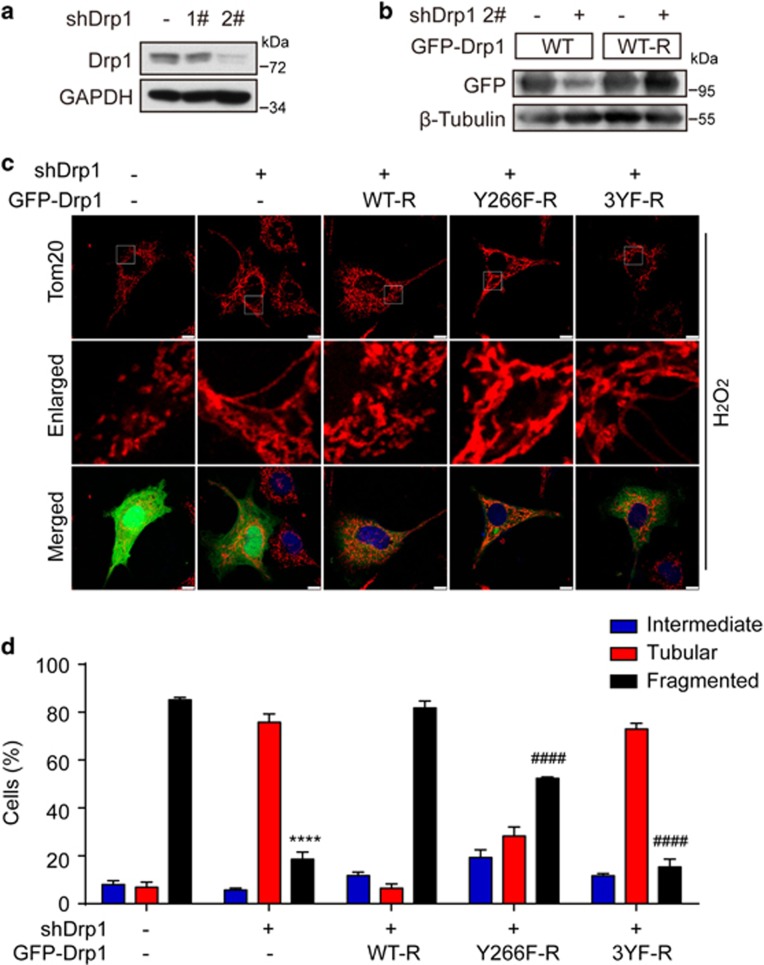
c-Abl mediated Drp1 phosphorylation is associated with mitochondrial fragmentation. (**a**) Lysates of SH-SY5Y cells infected with lentivirus of Drp1 knockdown were immunoblotted with anti-Drp1 or GAPDH antibody. (**b**) Lysates of HEK 293T cells transfected with the indicated plasmids were immunoblotted with anti-GFP or *β*-tubulin antibody. (**c**) SH-SY5Y cells transfected with the indicated plasmids and treated with 100 *μ*M H_2_O_2_ for 3 h were immunostained with anti-Tom20 antibody and DNA dye DAPI, followed by confocal microscopy. Scale bars, 10 *μ*m. (**d**) Mitochondrial morphology was assessed from 80 to 120 cells on three different slides. (two-way ANOVA, fragmented: ****P<0.0001, compared with shRNA vector+pEGFP vector group; ^####^*P*<0.0001, compared with shDrp1+GFP-Drp1 WT-R group, *n*=3 slides)

**Figure 7:**
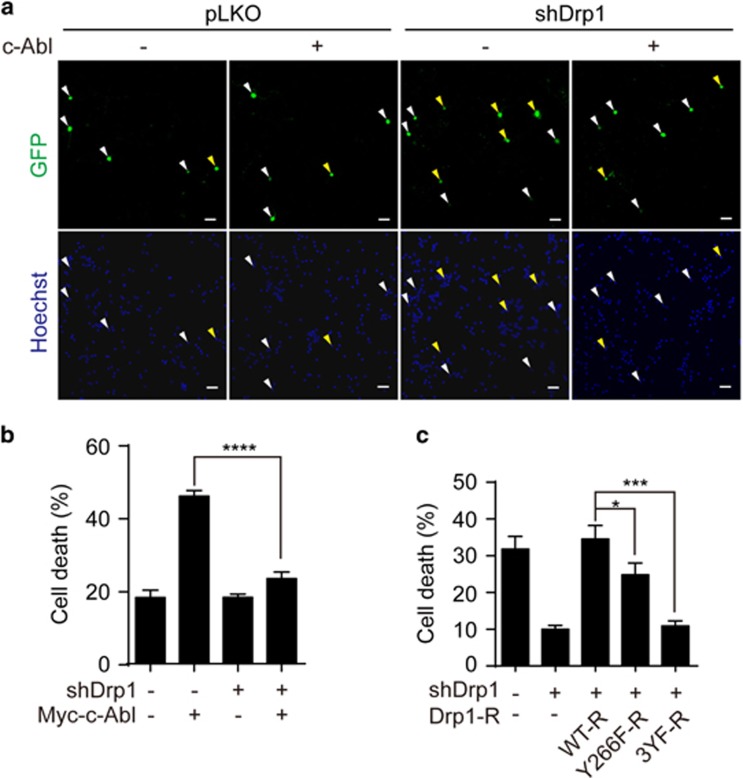
Drp1 phosphorylation mediated by c-Abl promotes neuronal cell death. (**a**) The DIV4 cortical neurons were transfected with pEGFP, alone or together with the Myc-c-Abl expression plasmid, and Drp1 RNAi (shDrp1 2#) or control vector as indicated. Seventy-two hours later, the neurons were fixed and stained with Hoechst 33258 and cell death was counted. Apoptotic cells are denoted by yellow arrows, and surviving cells are denoted by white arrows. Scale bars, 50 *μ*m. (**b**) Statistical analysis of panel (**a**) (one-way ANOVA, ****P<0.0001, *n*=3). (**c**) The DIV4 cortical neurons were transfected with pEGFP and Drp1 RNAi or control vector, alone or together with Drp1 WT-R, Y266F-R and 3YF-R expression plasmids. Seventy-two hours later, the neurons were treated with 50–80 *μ*M H_2_O_2_ for 12 h and stained with Hoechst 33258 for cell death assay. (one-way ANOVA, *P<0.05, ***P<0.001, *n*=3)

Given that the phosphorylation mediated by c-Abl regulates GTPase activity of Drp1, we next evaluated the effect of Drp1 mutants on mitochondrial morphology and neuronal cell death under oxidative stress. To this end, we first reduced the levels of endogenous Drp1 using two individual Drp1 knockdown plasmids, which target two different coding regions of Drp1 (Figure 6a). Then we transfected Drp1 RNAi resistant plasmids in SH-SY5Y cells for the rescue experiments (Figure 6b). It has been reported that oxidative stress could induce excessive mitochondrial fission, which could be blocked by Drp1 knockdown.^13^ Consistently, we found that Drp1 knockdown significantly reduced oxidative stress-induced mitochondrial fragmentation (18.53±5.32%), and interestingly, re-introducing WT rescue-form Drp1 (WT-R) in cells markedly increased mitochondrial fragmentation (81.76±5.12%). However, rescue forms of Y266F-R or 3YF-R Drp1 showed low ability to induce mitochondrial fission under oxidative stress, 52.38±1.04% and 15.39±5.61%, respectively (Figures 6c and d). In contrast, under normal culture condition, Drp1 mutants (Y266F-R and 3YF-R) increased mitochondrial fusion, which might be due to certain levels of GTPase activity (Supplementary Figures S5A and B). Next, we tested the role of c-Abl-mediated phosphorylation of Drp1 in oxidative stress-induced neuronal cell death. It has been shown that overactivation of c-Abl or excessive mitochondrial fission would lead to neuronal damage.^7, 23, 33, 34^ As shown in Figures 7a and b, overexpression of c-Abl increased cell death in primary cortical neurons, which could be inhibited by Drp1 knockdown, indicating that c-Abl and Drp1 shared a signaling cascade to regulate the neuronal cell death in response to oxidative stress. Furthermore, we found that Drp1 Y266F-R or Drp1 3YF-R expression could significantly inhibit oxidative stress-induced neuronal cell death compared with Drp1 WT-R in the background of Drp1 knockdown (Figure 7c). Taken together, c-Abl-mediated Drp1 phosphorylation promoted oxidative stress-induced neuronal cell death.

## Discussion

**Figure 8:**
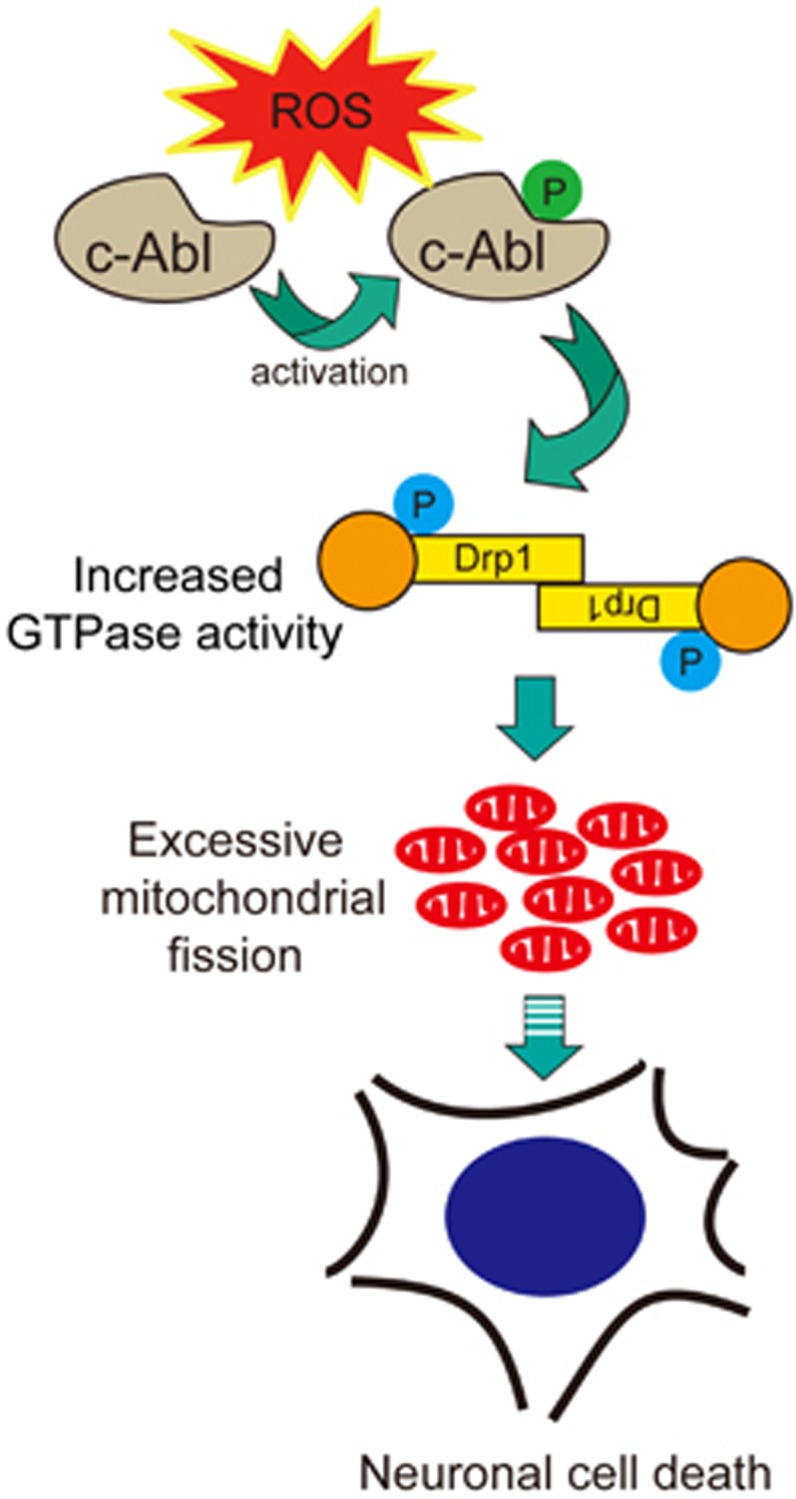
Working model by which oxidative stress regulates neuronal cell death via c-Abl-Drp1 signaling. In this model, activated c-Abl induces Drp1 tyrosine phosphorylation, which augments its GTPase hydrolysis activity and promotes Drp1-mediated mitochondrial fragmentation in response to oxidative stress. Mitochondrial fragmentation is involved in the initiation of apoptosis, finally leading to neuronal cell death

In the present study, we described a novel mechanism that c-Abl regulates mitochondrial morphology and oxidative stress-induced neuronal cell death by targeting Drp1 (Figure 8). c-Abl promotes mitochondrial fission through the interaction and phosphorylation of Drp1 at Y266, Y368 and Y449 under oxidative stress. Pharmacological inhibition or genetic deletion of c-Abl attenuates tyrosine phosphorylation of Drp1 (Figure 2d and Figures 3b–d) and rescues the mitochondrial morphological defects (Figure 1a and Supplementary Figures S1A–C). We thereby establish that c-Abl-mediated Drp1 phosphorylation increased mitochondrial fragmentation and induced neuronal cell death upon oxidative stress, indicating that c-Abl-Drp1 signaling cascade might have an important role in the pathogenesis of PD.

Multiple lines of evidence indicated that c-Abl has an essential role in the regulation of neuronal cell death. Many proteins have been identified as substrates of c-Abl, especially in PD.^25, 26, 27, 35, 36, 37, 38^ However, the relationship between c-Abl and mitochondria in PD is poorly studied. Kumar *et al.*^39^ have reported that PKC*δ* activates c-Abl, targeting it to mitochondria where it participates in initiating apoptosis by an unclear mechanism. Here we have clearly proved mitochondrial fission protein Drp1 as a novel substrate of c-Abl in the process of oxidative stress-induced mitochondrial fragmentation and neuronal cell death. Whether PKC*δ* is involved in c-Abl-Drp1 signaling pathway needs further investigation.

It has been reported the posttranslational phosphorylation of Drp1 have important roles in mitochondrial dynamics and cell fate determination.^3, 14, 21, 40^ For example, PKA-mediated Drp1 phosphorylation at Ser^637/656^ attenuates the GTPase activity of Drp1 and promotes cell survival. However, phosphorylation of Drp1 Ser^616^ by CDK5 or PKC*δ* induces its translocation to mitochondria, increase mitochondrial fragmentation and promotes cell death. In our experiments, c-Abl KO mitigated MPTP-induced both tyrosine and Ser^616^ phosphorylation in brain tissues (Figures 1c and d and Figures 3c and d) and c-Abl activation also increased Ser^616^ phosphorylation (Figure 1g and Supplementary Figure S1D), indicating that a crosstalk may exist between Ser^616^ and tyrosine phosphorylation in the regulation of Drp1 function. However, tyrosine-to-phenylalanine mutations of Drp1 failed to alter Ser^616^ phosphorylation in the overexpression experiments. Furthermore, serine-to-alanine mutation of Drp1 could not affect c-Abl-mediated tyrosine phosphorylation and GTPase activity (Supplementary Figures S4A and B). This data suggest that there is no interplay between tyrosine and Ser^616^ phosphorylation of Drp1 protein. Together, these results argue that there are two parallel signaling events in the regulation of mitochondrial fragmentation and cell death: one is c-Abl-mediated direct tyrosine phosphorylation and GTPase activation of Drp1, and the other is c-Abl-induced indirect Ser^616^ phosphorylation and mitochondrial translocation of Drp1.

Mitochondrial morphology is dynamically regulated through the fission and fusion balance, which is dependent on the activation of Drp1, including mitochondrial localization, self-assembly and GTPase activity. Altered GTPase activity of Drp1 is usually found to cause mitochondrial dysfunction in human diseases, especially in neurodegenerative diseases. Cho *et al.*^18^ have reported that *β*-amyloid protein stimulates NO production and cause *S*-nitrosylation of Drp1 at Cys^644^ within the GED domain, which enhances GTPase activity and Drp1 oligomerization in association with excessive mitochondrial fission in the brains of Alzheimer’s disease patient. Additionally, Song *et al.*^2^ have also reported that mutant huntingtin protein interacts with Drp1 and increases its GTPase activity in Huntington’s disease patient and mouse models. Therefore, our results argue that c-Abl-mediated Drp1 phosphorylation increased its GTPase activity and c-Abl-Drp1 signaling have a key role in the pathogenesis of neurodegenerative diseases.

In summary, our study revealed that c-Abl phosphorylated Drp1 and promoted Drp1-mediated mitochondrial fragmentation by upregulating its GTPase activity. Furthermore, inhibition of Drp1 phosphorylation mediated by c-Abl attenuated oxidative stress-induced cell death in primary cortical neurons, suggesting that c-Abl-Drp1 signaling pathway might be a potential target for the treatment of neurodegenerative diseases.

## Materials and methods

### Plasmids and transfection

The plasmids used were as follows: pCMV-Myc-c-Abl WT and KD were as previously described.^23^ The hDrp1 fragments were cloned into pCMV-3xFlag and pEGFP-C2 expression vectors. The EGFP-tagged Drp1 constructs were inserted into pQCXIH vector. The Y266F, Y368F and Y449F mutants of Drp1 were generated via site-directed mutagenesis. All mutations were verified via sequencing. His-sumo tagged Drp1 fragments were cloned into pET-28a (+) vector. All small hairpin RNA (shRNA) fragments were inserted to the pLKO.1 vector between its EcoRI and AgeI sites. The targeting sequence of each construct is listed as follows: shDrp1-1#: GAGTGTAACTGATTCA; shDrp1-2#: AAGCAGAAGAATGGGGTAAAT. Unless stated, all transfections were performed in complete medium with Lipofectamine 2000 (Invitrogen, Waltham, MA, USA) or Vigofect (Vigorous Biotechnology, Beijing, China) according to the manufacturer’s protocols. Cells were collected at 24–36 h after transfection for overexpression assays or 72 h for knockdown assays.

### Animals

All the mice were maintained under conditions of a 12-h light/dark cycle at 23 °C and were provided with food and water *ad libitum* in the Animal Care Facility at the Institute of Biophysics, Chinese Academy of Sciences (Beijing, China). The generation of mice with a conditional c-Abl KO in neurons was performed as described previously.^24^

### Drugs

The following drugs were purchased: STI571 from Selleckchem (Houston, TX, USA), and H_2_O_2_ and MPTP from Sigma-Aldrich (St. Louis, MO, USA).

### Cell culture

HEK 293T and SH-SY5Y cell lines were cultured in high-glucose DMEM (Invitrogen) supplemented with 10% fetal bovin serum (Gibco, Grand Island, NY, USA), 50 U/ml penicillin and 50 *μ*g/ml streptomycin, in 5% CO_2_ atmosphere at 37 °C. Primary cortical neurons culture and subsequent cell death assay were performed as described previously.^41^

### Co-immunoprecipitation and immunoblotting

Co-immunoprecipitation and immunoblotting were performed as described previously.^24^ The following antibodies were used: polyclonal rabbit anti-GFP (A11122, Invitrogen), monoclonal mouse anti-Flag (F3165, Sigma), monoclonal mouse anti-Myc (Sc-40, Santa Cruz Biotechnology, Santa Cruz, CA, USA), monoclonal mouse anti-phospho-tyrosine (05-321, Millipore, Billerica, MA, USA), polyclonal rabbit anti-Tom20 (Sc-11415, Santa Cruz), polyclonal rabbit anti-c-Abl (#2862s, Cell Signaling Technology, Cambridge, MA, USA), polyclonal rabbit anti-phospho-c-Abl (Tyr-245) (#2861, Cell Signaling Technology), monoclonal mouse anti-DRP1 (611112, BD, San Jose, CA, USA), monoclonal mouse anti-*β*-Tubulin (CW0098A, CWbiotech, Beijing, China), and anti-VDAC1 (ab15895, Abcam, Cambridge, MA, USA).

### Immunofluorescence

Freshly fixed cells were washed with phosphate-buffered saline (PBS) three times and blocked with 20% goat serum in PBS containing 0.2% TritonX-100 for 1 h at room temperature. Cells were then incubated with the primary antibody at 4 °C overnight. After washing with PBS four times, Alexa Fluor 488- or 546-conjugated secondary antibody (Invitrogen) was used to detect the signal. The secondary antibody was incubated for 1 h at room temperature, and then nuclear morphology was visualized using the Hoechst 33258 or DAPI (Sigma).

### Immuno-electron microscopy

Mice were transcardially perfused with 4% PFA in PBS, pH 7.4. Perfused brains were postfixed and then cryo-protected gradually up to 30% sucrose (Sigma). Tissue was then cut into 50-*μ*m-thick coronal sections using a cryostat. Cryostat sections were blocked in 5% normal goat serum, 1% BSA and 0.3% TritonX-100 in PBS for 1 h at room temperature. Section was then incubated with polyclonal anti-TH antibody (1 : 200, P40101, Pel Freez Biologicals, Rogers, AR, USA) for three nights at 4 °C, followed by biotinylated goat anti-rabbit for two nights. Sections were then incubated in streptavidin-conjugated HRP (Vectastain ABC Kit, Vector Laboratories, Burlingame, CA, USA) for one night at 4 °C before being reacted with 3,30-diaminobenzidine. After immune-detection, the sections were postfixed with 2.5% glutaraldehyde overnight followed by 1% osmium tetroxide for 2 h. After dehydration and infiltration, sections were embedded in Spi-pon812 resin and polymerized and then sectioned with microtome (Leica EM UC6, Vienna, Austria). The ultrathin sections were collected on copper grids, stained with uranyl acetate and lead citrate and examined by transmission electron microscopy (FEI Tecnai Spirit 120kv, Hillsboro, OR, USA). The analysis was performed on digital images obtained from CCD camera. The quantification of mitochondrial size was performed with the examiner blind to the genotype and treatment using ImageJ 1.43u (NIH, Bethesd, MD, USA).

### Analysis of mitochondrial morphology

Cultured primary neurons or SH-SY5Y cells were fixed with 4% paraformaldehyde in PBS for 10 min and mounted. Immunofluorescence images were obtained using a 60 objective lens by Nikon confocal microscope (Nikon, Melville, NY, USA). Neurons or SH-SY5Y cells containing fragmented (small round dots), intermediate or tubular (excessive fused, elongated) mitochondria were defined as fragmented, intermediate or tubular, respectively, and analyzed in a single blind trial by an observer blind to the treatment status of cells to exclude the observer bias. Mitochondrial length in striatum and SN was measured using ImageJ 1.43u (NIH).

### Dissection and tissue extraction

Mice were killed by cervical dislocation at 2 h after the final MPTP injection and the brains were quickly dissected on ice. Striatum and ventral midbrain were localized with the aid of a mouse atlas. The tissue samples were immediately frozen in liquid nitrogen and stored at −80 °C until extraction. At extraction, the tissue samples were immersed in appropriate volumes of cell lysis buffer and disintegrated by vigorous mixing for 1 min using a precooled homogenizer. Then the tissue samples were centrifuged (12 000 r.p.m. at 4 °C for 10 min) after lysis for 10 min on ice and the supernatants were collected and quantified for western blotting analysis.

### *In vitro* kinase assay

Recombinant active c-Abl kinase (Millipore) was incubated for 30 min at the following reaction conditions: 20 mM Tris (pH 7.4), 10 mM MgCl_2_, 100 *μ*M ATP, and 1 *μ*g His-sumo-Drp1 or anti-Flag antibody immunoprecipitates from Flag-Drp1-transfected 293T cells as substrate. Kinase reactions were separated by SDS-PAGE gel electrophoresis and analyzed by immunoblotting with the indicated antibodies.

### GTPase assay

A total of 1 mg whole-cell extract was immunoprecipitated for 3 h with anti-Flag M2 affinity gel. After three washes with lysis buffer and three washes with GTPase buffer (50 mM Tris (pH 7.4), 2.5 mM MgCl_2_ and 0.02% 2-mercaptoethanol), the beads were incubated with 0.5 mM GTP at 30 °C for 1 h. The released free phosphate was quantified using the PiColorLock Gold Kit from Innova Biosciences (Babraham, Cambridge, UK).

### Statistics

All values are expressed as mean±S.E.M. *P<0.05, **P<0.01 and ***P<0.001 denote the significance thresholds. Statistical analysis of the data was carried out with two-tailed Student’s *t*-test for two groups or one-way or two-way ANOVA for multiple groups.
