# Supplementary figures and images for: c-Abl-mediated Drp1 phosphorylation promotes oxidative stress-induced mitochondrial fragmentation and neuronal cell death
Source: Cell Death Dis. 2017 Oct 12;8(10):e3117–. doi: 10.1038/cddis.2017.524 (PMC5682686; doi:10.1038/cddis.2017.524)

Figure S1

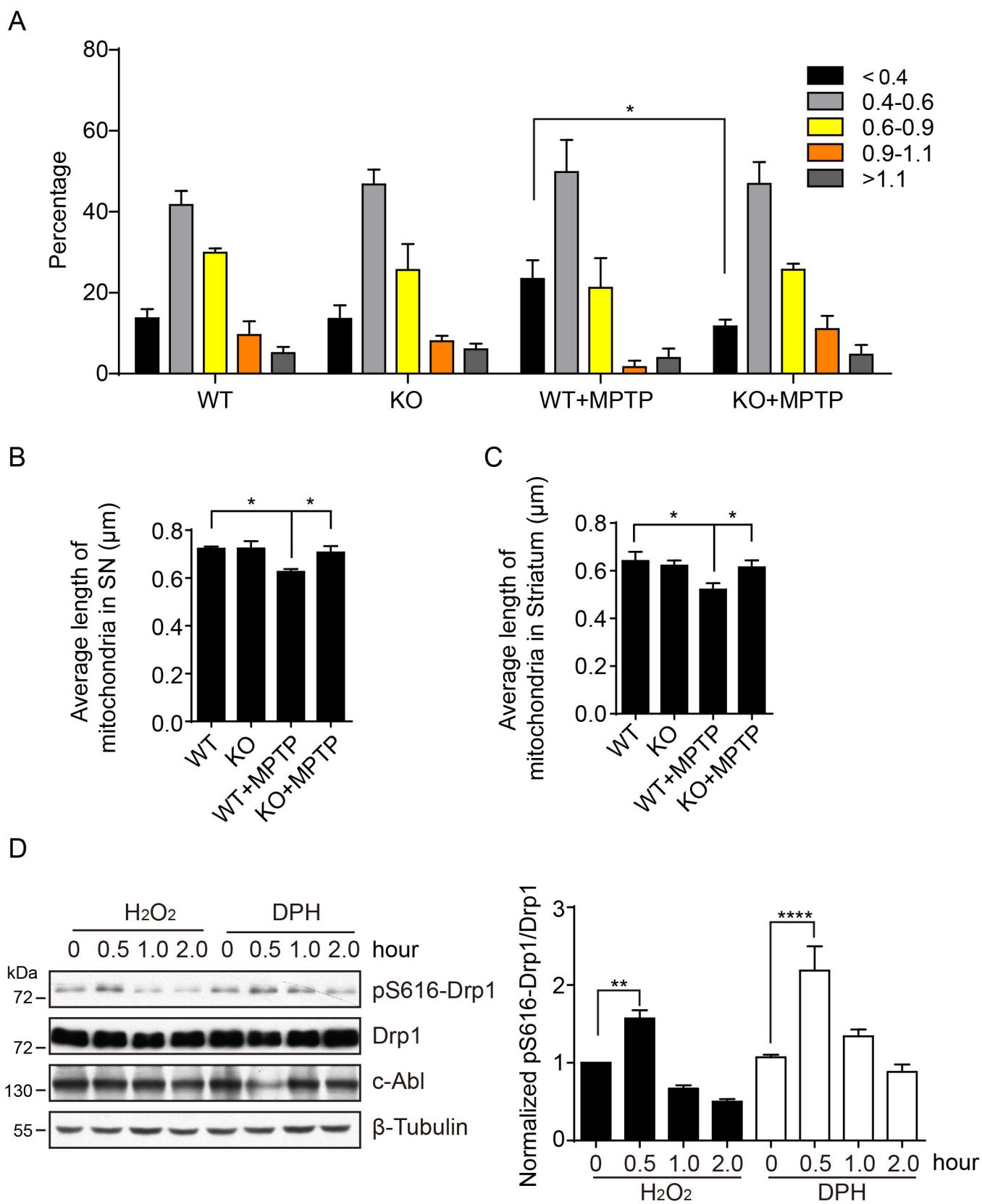

Supplement: Supplementary Figure S1 [file cddis2017524x1.pdf]

Figure S2

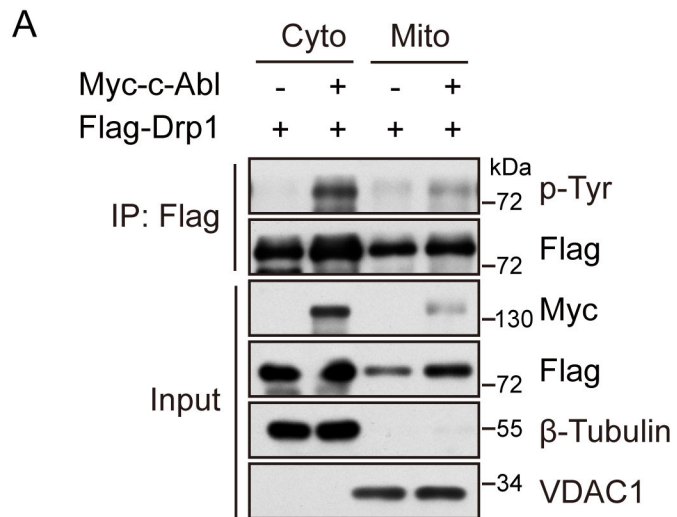

Supplement: Supplementary Figure S2 [file cddis2017524x2.pdf]

Figure S3

A

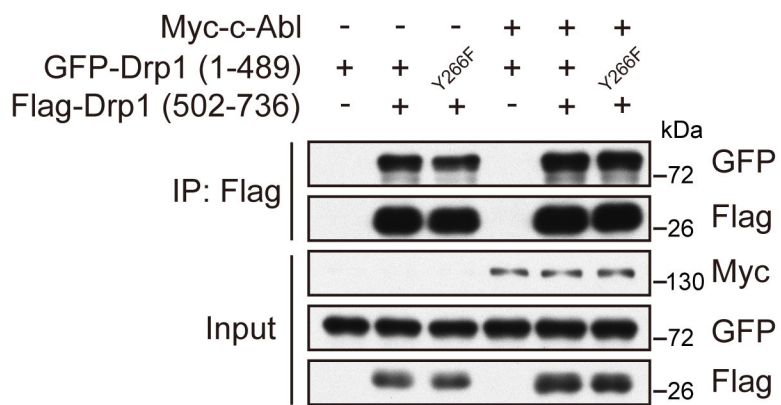

B

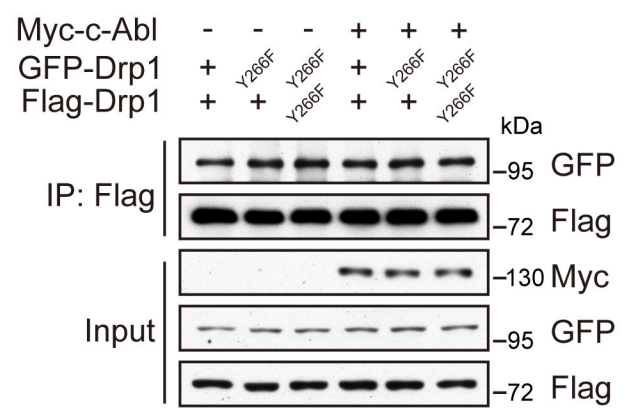

Supplement: Supplementary Figure S3 [file cddis2017524x3.pdf]

Figure S4

A

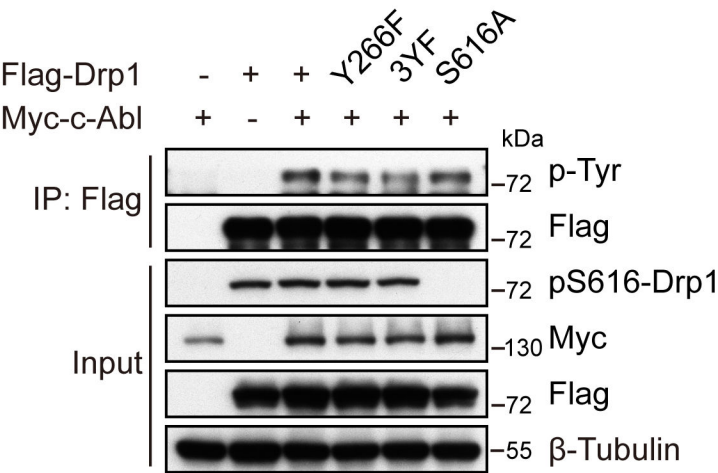

B

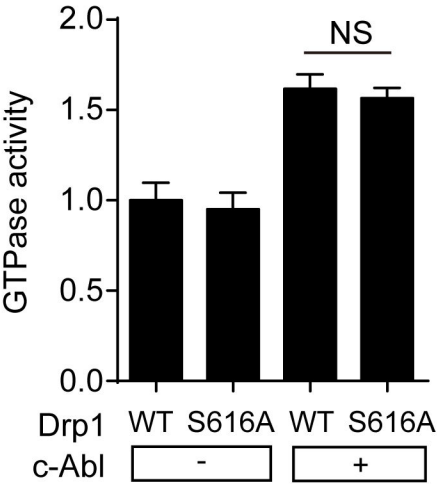

Supplement: Supplementary Figure S4 [file cddis2017524x4.pdf]

Figure S5

A

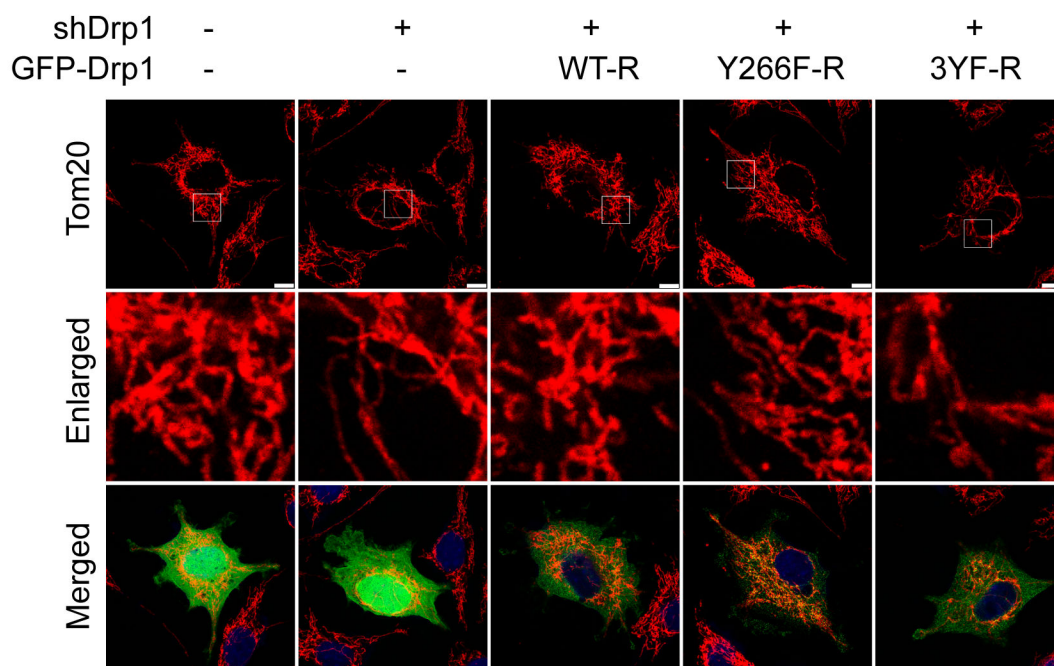

B

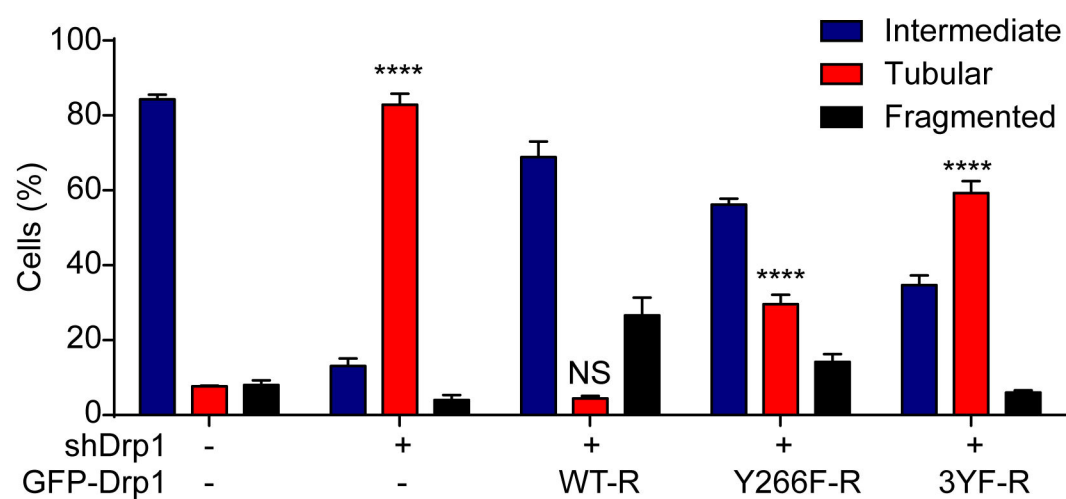

Supplement: Supplementary Figure S5 [file cddis2017524x5.pdf]
