# Supplementary material for: c-Abl-mediated Drp1 phosphorylation promotes oxidative stress-induced mitochondrial fragmentation and neuronal cell death
Source: Cell Death Dis. 2017 Oct 12;8(10):e3117–. doi: 10.1038/cddis.2017.524 (PMC5682686; doi:10.1038/cddis.2017.524)
Supplement: Supplementary Figure Legends [file cddis2017524x6.docx]

**Supplementary Information**

**Figure S1. c-Abl promotes mitochondrial fission *in vivo***

(A) Mitochondrial length in TH positive neurons of striatum was measured and binned into different categories of < 0.4, 0.4-0.6, 0.6-0.9, 0.9-1.1 and > 1.1 µm. Quantification of mitochondrial length are shown as percentages of total mitochondria. (Two-way ANOVA, *P < 0.05, n=3 mice)

(B and C) Average length of mitochondria was calculated in SN (B) and striatum (C).

(D) Left panel: lysates of SH-SY5Y cells treated with 500 μM H_2_O_2_ or 0.5 μM DPH for indicated time were immunoblotted with indicated antibodies. Right panel: The normalized levels of pS616-Drp1. (One-way ANOVA, ** p < 0.01, **** p < 0.0001, n=3)

**Figure S2. Distribution of Drp1 phosphorylation by c-Abl**

(A) Lysates of cytosolic and mitochondrial fraction of HEK 293T cells transfected with Flag-Drp1, with or without c-Abl, were immunoprecipitated with anti-Flag antibody and analyzed by immunoblotting anti-phosphotyrosine antibody.

**Figure S3. Drp1 phosphorylation by c-Abl does not affect intra/inter-molecular interaction of Drp1**

(A) HEK 293T cells were transfected with GFP-Drp1 (1-489) and Flag-Drp1 (502-736), with or without Myc-c-Abl. At 24 hour after transfection, cells were lysed and subjected to immunoprecipitation with anti-Flag antibody, then immunoblotted with anti-GFP antibody.

(B) HEK 293T cells were transfected with GFP-Drp1 and Flag-Drp1, with or without Myc-c-Abl. At 24 hour after transfection, cells were lysed and subjected to immunoprecipitation with anti-Flag antibody, then immunoblotted with anti-GFP antibody.

**Figure S4. There is no mutual interference between tyrosine phosphorylation and Ser^616^ phosphorylation of Drp1**

(A) HEK 293T cells were transfected with Flag-Drp1 or mutants, with or without Myc-c-Abl as indicated. At 24 hour after transfection, cells were lysed and subjected to immunoprecipitation with anti-Flag antibody, then immunoblotted with anti-phosphotyrosine antibody and pS616-Drp1 antibody.

(B) Immunoprecipitated Drp1 WT or S616A transfected with or without Myc-c-Abl in HEK 293T were subjected to GTPase assay. (One-way ANOVA, ****P < 0.0001, n = 3)

**Figure S5.** **Drp1 mutants affect mitochondrial morphology**

(A) SH-SY5Y cells transfected with indicated plasmids were immunostained with anti-Tom20 antibody and DNA dye DAPI, followed by confocal microscopy. Scale bars, 10 μm.

(B) Mitochondrial morphology was assessed from 80-120 cells on three different slides. (Two-way ANOVA, tubular: ****P < 0.0001, compared with shRNA vector + pEGFP vector group, n = 3 slides)
